# Acute Pericarditis-Induced Brugada Phenocopy: A Case Report and Review of the Literature

**DOI:** 10.7759/cureus.9761

**Published:** 2020-08-15

**Authors:** Malik Shehadeh, Susan O'Donoghue

**Affiliations:** 1 Internal Medicine, MedStar Washington Hospital Center, Washington, USA; 2 Electrophysiology, MedStar Heart and Vascular Institute/MedStar Washington Hospital Center, Washington, USA

**Keywords:** brugada syndrome, brugada phenocopy, brugada pattern, pericarditis

## Abstract

Brugada phenocopies are interesting clinical entities with electrocardiographic (ECG) patterns indistinguishable from the inherited Brugada syndrome. In patients with Brugada phenocopies, these ECG patterns are expected to resolve with resolution of the underlying condition.

## Introduction

Brugada syndrome is an inherited cardiac channelopathy associated with a high risk of syncope and sudden cardiac death due to serious heart arrhythmias [1]. Patients with Brugada syndrome have characteristic electrocardiographic (ECG) patterns in the right precordial leads [1,2]. However, similar ECG patterns have been reported in a variety of other conditions, known as Brugada phenocopies, despite a lack of underlying genetic defects [2,3]. In Brugada phenocopies, the Brugada ECG pattern is expected to disappear within a few days of withdrawal of the provoking factor.

We report a case of a patient with acute pericarditis who developed a Brugada ECG pattern that completely resolved with the treatment of the underlying pericarditis.

## Case presentation

A 35-year-old male patient without a significant medical history presented with substernal chest pain of one-day duration. The patient described his substernal chest pain as radiating to his back and worsening with inspiration and leaning forward. His history was remarkable for having a cold two weeks prior to his presentation.

Upon arrival to the hospital, his vital signs were unremarkable. Laboratory investigations showed normal while blood counts, normal lipid profile, and normal hemoglobin A1c. A chest X-ray didn’t show any evidence of pneumonia. A chest CT angiography was negative for pulmonary embolus or aortic dissection.

**Figure 1:**
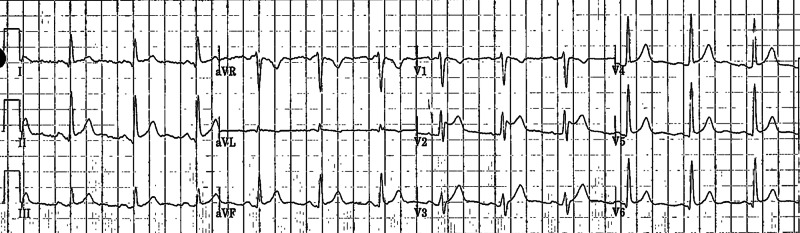
ECG on presentation ECG showing diffuse ST-PR discordance secondary to acute pericarditis along with type II Brugada pattern, which manifested in lead V2 as a saddle-back pattern with at least 2-mm J-point elevation and at least 1-mm ST elevation with a positive T–wave ECG, electrocardiogram

Two assays of troponin were performed six hours apart and were negative. His ECG showed diffuse ST-PR discordance, which was highly concerning of pericarditis, along with type II Brugada pattern, which manifested in lead V2 as a saddle-back pattern with at least 2-mm J-point elevation and at least 1-mm ST elevation with a positive T-wave (Figure 1). An echocardiogram was performed and showed normal left ventricular ejection fraction of 55% without regional wall motion abnormality or pericardial effusion.

**Figure 2:**
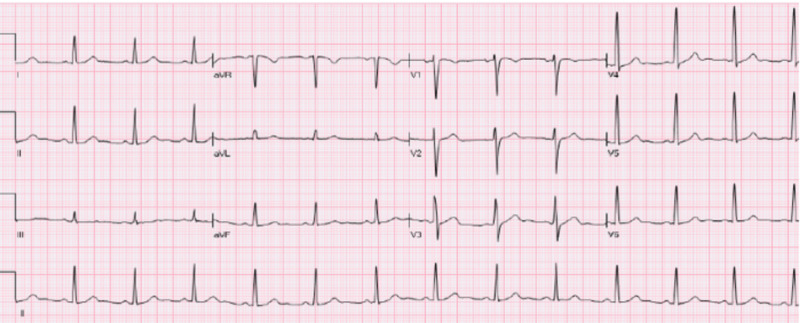
ECG after two weeks of treatment Complete resolution of the type II Brugada pattern was noticed with resolving pericarditis after two weeks of treatment. ECG, electrocardiogram

Given the history, clinical picture, and lack of family history of arrhythmias or sudden cardiac death, his ECG features in lead V2 were interpreted as Brugada phenocopy rather than Brugada syndrome. The patient was treated as a case of post-viral pericarditis. He was started on nonsteroidal anti-inflammatory drugs and colchicine, which lead to significant improvement in his symptoms and complete resolution of ECG features within two weeks (Figure 2) (Poster: Malik Shehadeh, John Costello, Vijaywant Brar, Susan O'Donoghue. Acute Pericarditis Mimicking Brugada Syndrome. Society of General Internal Medicine, May 2020, Birmingham, AL, USA).

## Discussion

Brugada syndrome is an autosomal dominant genetic disorder with incomplete penetrance characterized by disturbances affecting the electrical activity within the heart. Patients with Brugada syndrome might experience syncope or sudden cardiac death due to serious abnormal heart rhythms such as ventricular fibrillation. Genetic analysis revealed that most of the causative mutations involve the cardiac sodium channel genes [1,4]. More than 100 mutations have been described so far in the literature [1]. The relationship between the sodium channel abnormalities and characteristic ECG patterns seen in the right precordial leads in patients with Brugada syndrome is not fully understood.

The ECG features for patients with Brugada syndrome can be divided into two distinct patterns: one with a coved-type ST segment elevation (type I Brugada pattern) and the other one with a saddle-back ST segment elevation (type II Brugada pattern).

Diagnosis of Brugada syndrome requires a characteristic type I Brugada pattern and clinical findings (i.e., arrhythmia, syncope, sudden cardiac death). However, some patients may be diagnosed based on relevant family history of sudden cardiac death or coved-type ECGs in family members. In patients with type II Brugada pattern, diagnosis is considered positive when conversion to the diagnostic type I Brugada pattern occurs after a sodium channel blocker drug challenge test [1-3]. Once diagnosis is made, these patients will need follow-up on a regular basis and family screening in first-degree relatives, and might benefit from an implantable cardioverter-defibrillator (ICD) placement.

The term “Brugada phenocopy” is proposed to describe clinical conditions with Brugada ECG patterns in patients without underlying genetic defect or family history of sudden cardiac death. There are hundreds of published cases of Brugada phenocopies due to various clinical conditions such as metabolic derangements, myocardial ischemia, infiltrative cardiomyopathy, pericardial disease, mechanical mediastinal compression, and pulmonary embolism [3]. Aiming the clarification of this topic, the International Registry of Brugada phenocopies maintains an online database for the documentation of these patients. It is crucial to recognize that in all these clinical entities, the Brugada ECG patterns are expected to resolve with resolution of the underlying condition [3].

Only five cases of pericarditis-induced Brugada phenocopy have been reported in the literature [5-8]. Three of these cases presented with type II Brugada pattern and the other two presented with type I Brugada pattern (Table 1). In all these patients, the ECG patterns completely resolved with resolution of underlying pericarditis.

**Table 1:** Reported cases of pericarditis-induced Brugada phenocopy in the literature

Study	Patient age	Patient gender	Brugada ECG pattern
Ozeke et al. [5]	28	Male	Type II
	36	Male	Type II
Hermida et al. [6]	44	Male	Type I
Monti et al. [7]	27	Male	Type II
Yu et al. [8]	24	Male	Type I

We reported the sixth case of acute pericarditis-induced Brugada phenocopy. Our patient presented with Brugada-type II pattern and his ECG features resolved completely with resolution of underlying pericarditis.

## Conclusions

Acute pericarditis may mimic Brugada syndrome. The ECG patterns of Brugada syndrome shouldn’t be considered as a specific marker of the syndrome but rather as a sign of electrical heart disease that can be due to various other conditions. To make a definitive diagnosis in patients with a high clinical pretest probability of Brugada syndrome, further workup such as drug challenge with sodium channel blocker and genetic analysis might be required.
